# Open-World Games’ Affordance of Cognitive Escapism, Relaxation, and Mental Well-Being Among Postgraduate Students: Mixed Methods Study

**DOI:** 10.2196/63760

**Published:** 2024-12-17

**Authors:** Ailin Anto, Arunima Basu, Rania Selim, Thomas Foscht, Andreas Benedikt Eisingerich

**Affiliations:** 1 Faculty of Medicine Imperial College London London United Kingdom; 2 Department of Marketing The University of Graz Styria Austria; 3 Imperial College Business School Imperial College London London United Kingdom

**Keywords:** open-world games, cognitive escapism, relaxation, mental well-being, students, video games, stress, freedom to explore

## Abstract

**Background:**

Open-world games, characterized by their expansive and interactive environments, may offer unique cognitive escapism opportunities, potentially leading to relaxation and enhanced well-being. These games, such as “The Legend of Zelda: Breath of the Wild” and “The Legend of Zelda: Tears of the Kingdom,” allow players to experience a sense of freedom and autonomy, which can reduce stress and improve mental health. While previous research has examined the general impact of video games on mental well-being, specific studies on the effects of open-world games among postgraduate students are limited.

**Objective:**

This study aims to investigate the relationships between cognitive escapism provided by open-world games and their effects on relaxation and well-being. The goal was to understand how the immersive nature of these games contributes to stress reduction and overall mental health improvement among postgraduate students.

**Methods:**

A mixed methods approach was used, which involved in-depth exploratory qualitative interviews and a survey of 609 players of popular open-world games. Quantitative data were collected using standardized questionnaires to measure open-world games’ affordance of cognitive escapism, relaxation, and well-being. Qualitative data were obtained through 32 in-depth interviews that explored players’ experiences and perceptions of cognitive escapism, relaxation, and mental well-being.

**Results:**

Qualitative data (n=32; n=15, 47% female; n=16, 50% male; n=1, 3% preferred not to disclose gender; mean age 23.19, SD 2.19 y) revealed that cognitive escapism through immersive game worlds allowed players to temporarily disconnect from real-world stressors, resulting in enhanced mood and psychological well-being. Players indicated that the nonlinear gameplay and freedom to explore interactive environments provided a sense of relaxation and mental rejuvenation. Quantitative analysis (N=609) showed a substantial mediating role of relaxation in the relationship between cognitive escapism offered by open-world games and well-being. Specifically, cognitive escapism had a significant positive effect on players’ relaxation (β=.15; SE 0.04; *P*<.001; 95% CI 0.0695-0.2331), which in turn had a significant and positive effect on players’ well-being scores (β=.12; SE 0.04; *P*=.002; 95% CI 0.0445-0.2032).

**Conclusions:**

The study demonstrates that open-world games offer substantial benefits for cognitive escapism, significantly improving relaxation and well-being among postgraduate students. The immersive and autonomous nature of these games is crucial in reducing stress and enhancing mental health. Future research may investigate the long-term effects of regular engagement with open-world games and explore their potential therapeutic applications for managing stress and anxiety.

## Introduction

### Overview

Open-world games with their expansive, immersive environments and nonlinear gameplay have become a significant genre in the video game industry. Titles such as “The Legend of Zelda: Breath of the Wild” (Nintendo) or “The Legend of Zelda: Tears of the Kingdom*”* (Nintendo) provide players with computer-simulated environment to explore, fostering a sense of freedom, autonomy, and discovery. In this study, we explore the impact of open-world games on postgraduate students’ well-being by offering cognitive escapism and relaxation.

Today, an increasing number of young people report high levels of stress, anxiety, and sadness [1]. Prior research has demonstrated that video games can serve as effective tools for stress relief [2-4]. In particular, in this study, we posit that open-world games with their expansive environments and opportunities for leisurely exploration may create a sense of escapism and relaxation. Previous work found that casual video game play may significantly reduce stress and improve mood [5], suggesting potential benefits for players of open-world games, which often offer similarly engaging yet nonpressuring experiences. Moreover, prior research reviewed the cognitive benefits of action video games and found improvements in attention, spatial skills, and problem-solving abilities [6]. These cognitive benefits are likely applicable to open-world games due to their intricate gameplay and diverse challenges. For instance, open-world games often require players to solve complex puzzles, strategize, and make decisions that can enhance cognitive functions.

Cognitive escapism refers to the use of media to divert attention from real-world stressors and engage in alternative, often more gratifying, cognitive activities. Open-world games, with their detailed and expansive environments, may provide ideal platforms for this form of escapism. They allow players to immerse themselves in different realities, which can be mentally stimulating and refreshing. For instance, previous work on the psychological benefits of immersive virtual environments highlights the potential for such environments to offer significant cognitive relief [7].

In recent years, there have been some exploration of escapism in emerging contexts, including the metaverse [8-10] and recreational activities, such as gaming, web-based streaming, or running [4,11]. This study extends the work of previous research that has primarily focused on general gaming or specific genres, such as action or casual games [12-19]. By specifically focusing on open-world games, new insights can be gained about the nuanced ways these games facilitate cognitive escapism.

Specifically, the expansive nature of open-world games allows players to engage in non–goal-oriented activities, such as exploration, crafting, or simply enjoying the game’s scenery, which can foster relaxation. Unlike the high-intensity demands of many action games, open-world games often allow a more leisurely pace, contributing to relaxation and stress relief. Prior studies have shown that casual gaming can reduce stress. However, the open-world genre has been less frequently studied in this context. A focused study could, thus, provide robust evidence of how these games’ specific features, such as vast, interactive worlds and varied activity options, uniquely contribute to relaxation.

Despite the potential benefits of gaming, there is a risk of addiction associated with prolonged gaming [20]. Open-world games with their immersive nature may also lead to excessive play. The World Health Organization has recognized “gaming disorder” as a behavioral addiction, highlighting the need for moderation and awareness [21]. Furthermore, extended periods of gameplay can negatively affect physical health, contributing to a sedentary lifestyle. A prior study indicated that high levels of sedentary behavior are associated with increased risks of cardiovascular diseases and other health issues [22]. Players of open-world games, which often engage in extended play sessions, may balance their gaming with physical activity.

Thus, open-world games hold significant potential for enhancing well-being through stress reduction, cognitive skill development, and social interaction. However, the risks of addiction and physical health impacts must be carefully managed. Additional research is needed to explore the potential benefits of open-world games while addressing their potential harms. In this study, we aimed to explore the effects of open-world games on postgraduate students’ mental well-being. Students frequently operate in environments of stress and pressure [23,24]. Thus, we focus on examining the impact of open-world games in postgraduate students’ lives.

In brief, this study investigates the relationships between cognitive escapism provided by open-world games and their effects on relaxation and well-being. The goal was to understand how the immersive nature of these games contributes to stress reduction and overall mental health improvement among postgraduate students.

In the following sections, we provide background information on the study and examine the exploratory qualitative studies (study 1, n=17; study 3, n=15) and the quantitative cross-sectional survey (study 2, N=609), along with their findings. We conclude with a discussion of the research implications, limitations, and avenues for future research.

### Background on Open-World Video Games

**Figure 1:**
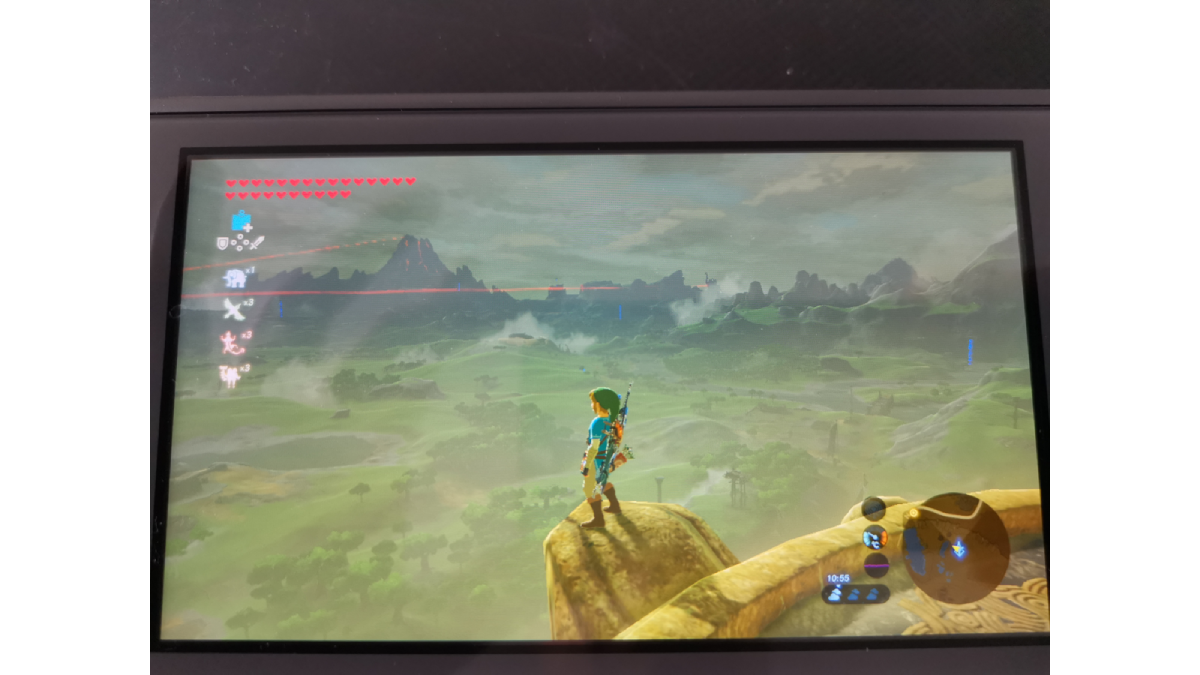
The open world of The Legend of Zelda: Breath of the Wild.

“Open-world” refers to games that provide a large, freely explorable environment, where players can move and interact with the game world with minimal restrictions. Here are some characteristics that make a game, such as Nintendo’s “Zelda: Breath of the Wild,” an open-world game. First, a key characteristic of open-world games is the freedom to explore; that is, players can roam the world at their own pace and choose their path and activities. Indeed, Shigeru Miyamoto shared that his main inspiration when creating the Zelda games series was to capture the essence of a young kid exploring outdoors, just as he did in the countryside of his hometown throughout his childhood. Moreover, open-world games often allow for nonlinear gameplay. Hence, the game does not force players to follow a set sequence of events or missions. Furthermore, they offer large, detailed worlds to explore. Specifically, the games may feature an expansive world filled with diverse landscapes, towns, and hidden secrets. For example, see Figure 1, which depicts Zelda’s protagonist, Link, in “The Legend of Zelda: Breath of the Wild”(Nintendo). Thus, by inviting exploration and discovery, open-world games provide a unique and engaging experience that contrasts with more competitive or goal-oriented gameplay. This approach allows players to immerse themselves in the game world, fostering a sense of wonder and curiosity.

Open-world games provide a unique gaming experience by creating vast, immersive environments that players can explore and discover at their own pace. This focus on exploration and discovery is distinct from traditional game objectives, such as competition or beating the game. More specifically, open-world games often feature large, meticulously designed environments filled with diverse landscapes, cities, villages, and natural wonders. These expansive settings encourage players to wander and investigate, providing a sense of freedom and autonomy. The detailed worlds often contain hidden secrets, lore, and environmental storytelling that reward curious players. Furthermore, unlike linear games with a set path, open-world games offer nonlinear gameplay. Players are not bound by a strict sequence of events and can choose their own path. This freedom allows them to engage with the game world on their own terms, whether they prefer to follow the main story, complete side quests, or simply explore.

The self-directed playstyle of open-world games promotes a deeper connection with the game world, with a primary focus on exploration. In contrast, competitive games, such as “Fortnite,” are structured around set objectives and a defined path. For instance, in the case of Fortnite, the primary goal is to be the last player or team standing, which drives a clear, competitive progression. Fortnite’s gameplay is fast paced and intense, designed to keep players constantly engaged and on their toes. The competitive nature drives a high level of excitement and urgency. Open-world games, in contrast, often emphasize player-driven experiences over predefined goals. This allows players to set their own objectives at their own pace and preference, whether it is building a new settlement, taming wild creatures, or mapping out uncharted territories.

In addition, open-world games offer a dynamic environment where the world responds to player actions and the time of day, with changes in weather, nonplayer characters’ behavior, and more. In addition to “Zelda: Breath of the Wild or Zelda: Tears of the Kingdom,” there are several popular video games that fall into the “open-world” category. Here are some notable examples across different genres and platforms: “The Elder Scrolls V: Skyrim” is a fantasy role-playing game that offers a vast world full of quests, dungeons, and a deep lore. “Red Dead Redemption” is a Western-themed action-adventure game with a highly immersive and realistic open world. “Minecraft” is a sandbox game that allows players to explore, build, and survive in a blocky, procedurally generated world. “Ghost of Tsushima” is an action-adventure game set in feudal Japan, known for its beautiful landscapes and samurai combat. These games, to name but a few, are known for their expansive, immersive worlds that offer players a high degree of freedom and numerous activities to engage in beyond the main storyline. Next, we discuss the potential effects of open-world games on players’ cognitive escapism, relaxation, and mental well-being.

### Open-World Video Games and Mental Well-Being

Well-being may encompass a wide range of psychological states, including happiness, stress levels, and overall mental health [25]. Open-world games, by providing opportunities for escapism and relaxation, can positively influence these aspects of well-being. The autonomy and control players experience in these games can also enhance feelings of competence and satisfaction. This study on well-being in relation to open-world games builds on existing literature that examines the psychological impacts of video games [26,27]. Previous research on the motivational aspects of video game engagement suggests that games fulfilling basic psychological needs can significantly enhance well-being [19]. Thus, a study on open-world games may further elucidate how the freedom and expansive environments in these games meet these psychological needs more effectively than other genres.

**Figure 2:**
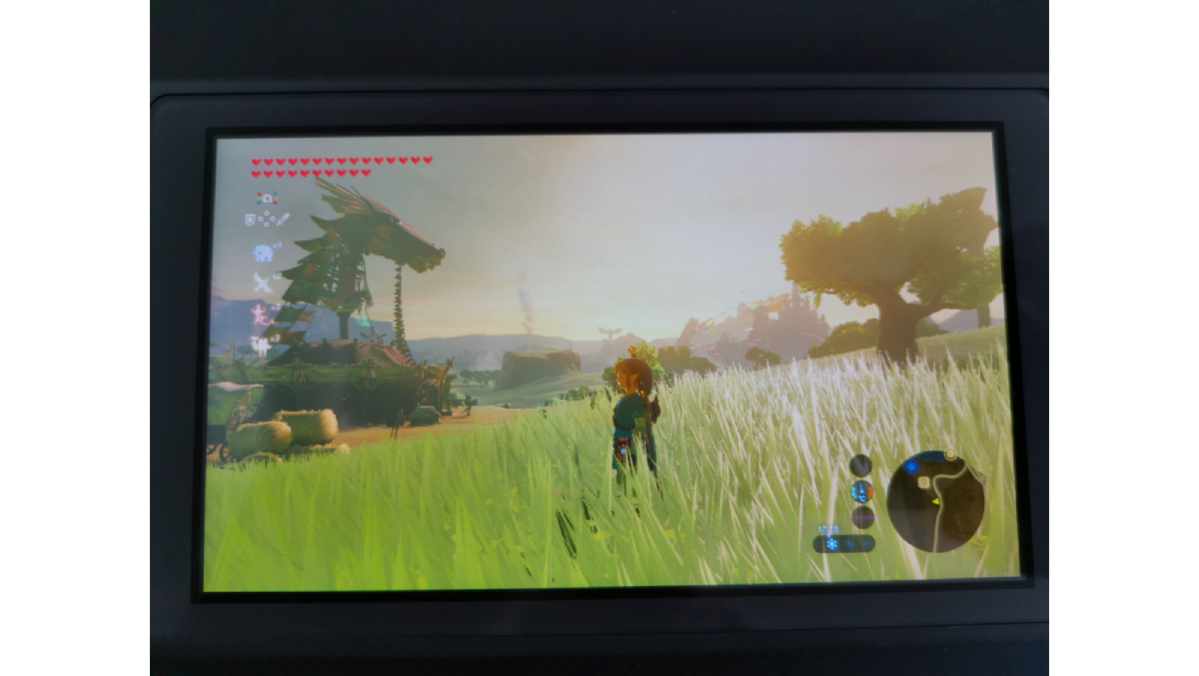
Link in The Legend of Zelda: Breath of the Wild, with parts of the Hyrule region in the background.

**Figure 3:**
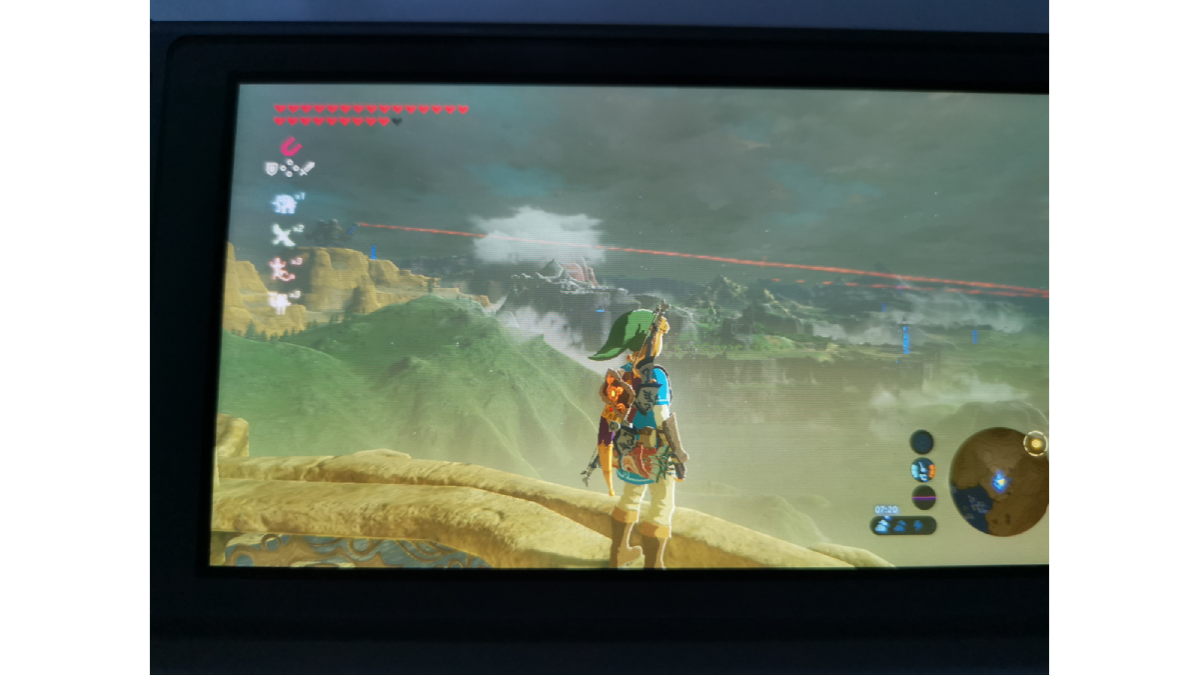
Link in The Legend of Zelda: Breath of the Wild, with parts of the Gerudo Highlands in the background.

Open-world games may provide a unique form of cognitive escapism by immersing players in expansive, interactive environments that divert their attention from real-world stressors. This immersive experience may lead to significant relaxation and improvements in overall well-being. Cognitive escapism refers to the process of diverting one’s thoughts away from real-life concerns to immerse in an engaging activity. Open-world games facilitate this through their richly detailed and varied worlds, compelling narratives, and freedom of exploration (Figures 2 and 3). The immersive nature of these games allows players to temporarily set aside their worries and become absorbed in an alternate reality. The process of cognitive escapism in open-world games can lead to profound relaxation.

According to a prior study, engaging in video games can help alleviate stress by providing a temporary escape from daily pressures, allowing the mind to rest and recuperate [12]. The nonlinear and often serene environments in open-world games, such as the tranquil landscapes in “The Legend of Zelda: Breath of the Wild,” enhance this effect by allowing players to explore and interact at their own pace without the immediate pressures of goal-oriented tasks.

Furthermore, prior work indicates that casual gaming may significantly reduce stress and improve mood [9]. The study highlights that engagement in low-pressure, enjoyable activities can lead to relaxation. Open-world games, with their extensive and varied environments, offer similar benefits by allowing players to choose their activities and engage in relaxing, noncompetitive gameplay. Cognitive escapism in open-world games may also provide emotional relief by allowing players to experience positive emotions and alleviate negative ones. Previous work found that immersive virtual environments could significantly improve mood and reduce anxiety [11], indicating that similar benefits could be derived from open-world game environments.

Well-being is a multifaceted construct that includes emotional, psychological, and social dimensions [25]. Cognitive escapism through open-world games may enhance well-being by providing mental stimulation, emotional relief, and opportunities for social interaction. The immersive experiences in open-world games can contribute to psychological well-being by fulfilling basic psychological needs, such as autonomy, competence, and relatedness, as described by self-determination theory [13]. The autonomy offered by open-world games allows players to make choices and control their in-game actions, which can lead to increased feelings of competence and satisfaction.

To explore the difference that playing an open-world game makes to students’ daily lives, we first conducted an exploratory study with qualitative in-depth interviews. The findings of the qualitative study informed the survey study and the second qualitative study. In the following sections, we discuss these studies.

## Methods

### Study 1

#### Qualitative Study Design

To generate novel insights from data gathered in a natural setting, we first conducted semistructured, cross-sectional interviews using open-ended, predetermined questions [28]. We report the findings of qualitative study 1 in accordance with the Standards for Reporting Qualitative Research guidelines [29]. This gave us a chance to explore and consider various personal views and opinions [30] and, in so doing, follow the spirit of theory in use approach [31]. Participants were invited to share their thoughts about open-world gaming and how it affects their daily lives. Specifically, during the interview, we asked respondents to describe how open-world gaming affects their daily lives.

As researchers with prior experience in qualitative studies, we were aware of the importance of in-depth data collection to formulate and derive new theories. We ensured that questions were open ended. Moreover, to minimize any potential bias, 2 trained research assistants (RAs) conducted the interviews, and any discrepancies were discussed. In addition to this, the 2 RAs were trained not to guide the participants and their responses in any shape or form. The team of RAs and the entire research team was diverse in gender, age, and ethnicity; this ensured increased comfortability with participants during interviews and subsequent discussions.

#### Ethical Considerations

Ethics approval for the study was given by Imperial College London at Departmental level, Analytics, Marketing, and Operations. The study was conducted in accordance with the principles of the Declaration of Helsinki. Informed consent was obtained, and participants had the right to withdraw from the study at any timepoint. Study data were anonymized and kept confidential within the research team. Participants were compensated US $30 for their time.

#### Participants and Procedure

We used a convenience sampling method and recruited study participants via posters and handing out flyers to students on a university campus. Eligibility criteria for taking part in the study was that they were full-time students and they had some personal experience playing an open-world game before. Over the course of 5 weeks (beginning of June 2024), 17 (n=9, 53% female; n=7, 41% male; n=1, 6% preferred not to disclose gender; mean age 22.88, SD 2.15 y) exploratory in-depth interviews were conducted with respondents, who were full-time students (student ID check) and have played an open-world game before (the trained RA asked some basic screening questions, including which specific open-world game or games they had play before and the basic content [story] of the games).

We followed established procedures for inductive qualitative data analysis when examining our qualitative data. Specifically, following constant comparison techniques in prior work [28], we collected and analyzed the interview data in tandem and compared the newly collected data against the existing insights. With this approach, we first aimed to group conceptually linked data and reduce them to a set of meaningful concepts [28]. First, we analyzed the data to develop a holistic understanding of respondents’ evaluations of open-world gaming and key elements of it that impact their lives. Furthermore, we used open coding and used descriptive phrases to name these codes to develop insights about key motivators and process explanations regarding respondents’ evaluation of open-world games and key elements thereof that may influence their mental well-being [32]. Finally, by integrating relevant literature and conducting joint discussions, we finalized the elements of our conceptual framework to reflect the key elements of open-game worlds that affect players’ well-being [28].

Interviews were conducted in the alumni lounge and a café of a university and lasted between 20 and 95 minutes. Interviewees were reassured of the strict confidentiality of their responses and were told that their participation would inform academic research. At the end of each interview, we gave interviewees a chance to ask any potential question that they had.

#### Qualitative Data Analysis

First, each RA familiarized themselves with the data collected by transcribing the interviews and reading through the transcripts. Second, the authors then analyzed the data to develop a holistic understanding of the qualitative data. We used open coding and used descriptive phrases to name these codes to develop insights about the potential effects of open-world games on users. Interview data coding was conducted in duplicate by the authors, and any discrepancies were discussed and resolved. We used the constant comparison technique and compared the newly collected interview data against the existing insights as we conducted more interviews. In doing so, our first goal was to group conceptually linked data to reduce them to a set of meaningful concepts. As a third step, we finalized the elements of our conceptual framework to reflect the key effects of open-world games on users’ well-being. This was done by grouping codes together under common ideas to derive overarching themes. Fourth, the themes were then revisited and reviewed by the researchers and compared with all the data collected to ensure that all key concepts had been captured by the key themes. Fifth and finally, findings were presented through a report that highlighted the key identified effects of open-world games with a description and exemplary quotes.

### Study 2

#### Survey Study Design

We used convenience sampling and a cross-sectional survey as part of study 2. Eligibility for this study required participants to be full-time university students with personal experience playing an open-world game. The study was conducted in June 2024. For this study, we followed the Strengthening the Reporting of Observational Studies in Epidemiology (STROBE) guidelines to report the observational, cross-sectional studies (Multimedia Appendix 1) [33].

#### Ethical Considerations

Ethics approval for study 2 was received from Imperial College London at Departmental level, Analytics, Marketing, and Operations. Data were anonymized and kept confidential within the research team. Participants provided informed consent and had the right to withdraw from the study at any time.

#### Participants and Procedure

We recruited study participants via posters and handing out flyers to students on a university campus during June 2024. Potential participants were informed that the study would take no longer than 20 minutes and that they would receive US $8 as a thank you for their participation. A total of 630 full-time university postgraduate students (ID check), who played an open-world game (2 trained RAs asked brief screening questions before the study, eg, which open-world game they had played and brief content or story of that game), were recruited to take part in this study over the course of 3 weeks. In total, 21 respondents did not complete the entire survey, and thus, the effective number of responses is 609.

In case participants played >1 open-world game, study participants were invited to think of the game that they played the most in terms of hours spent per week when answering the survey questions. This study was conducted in a lecture theater of a university and was supervised by 2 trained RAs. As part of the study, participants were invited to complete a brief paper-and-pencil questionnaire about their mental well-being. The questions were adapted from a previously published psychological well-being scale [34], which included items such as “I feel there is meaning to my present and past life,” “I have a sense of continued self-development and growth,” and “I have a positive attitude toward myself and life in general.” Participants also answered questions about the extent to which playing an open-world game provided relaxation, adapted from an established emotional relaxation scale [32]. These included statements such as “Playing an open-world game makes me feel content,” “When I play an open-world game, I feel relaxed,” and “Playing an open-world game makes me feel stressed” (reverse-coded). In addition, questions about cognitive escapism were included, such as “Playing an open-world game helps me escape from my everyday problems,” “Open-world games help me forget the troubles and anxiety in my life,” and “Playing open-world games makes me forget my worries.” Responses were measured on a 7-point Likert scale (1=strongly disagree and 7=strongly agree). No demographic data (gender, age, etc) were collected as part of this study. At the end of the study, each participant was thanked and received US $8 as compensation for their participation. In addition, participants had a chance to ask any questions that they may have.

#### Survey Data Analysis

Before testing the effects of open-world games’ affordance of cognitive escapism on relaxation and mental well-being, we evaluated the factor structure and the reliability scores for the measurement items in the study. Specifically, we followed best practices for conducting confirmatory factor analysis, and the results showed that all the measurement items significantly loaded on their intended factors (*P*<.001) with weak cross-loadings (<0.20) [35,36]. The average variance extracted were higher than the recommended threshold of 0.50, indicating the convergent validity among the items of each scale [36,37]. In support of discriminant validity of the constructs, the average variance extracted was greater than the squared correlations between constructs and the other variables [38]. Table 1 lists the specific measurement reliabilities and item factor loadings.

As part of this study, we also conducted a mediation analysis with the PROCESS macro [39] (model 4; bootstrapped samples=5000; 95% CI) to examine the effects of open-world games’ affordance of cognitive escapism (independent variable) on players’ relaxation (mediator) and, consequently, on players’ mental well-being (dependent variable).

**Table 1:** Measurement items’ factor loadings and reliabilities.

Construct and measurement items			Factor loadings
**Mental well-being (α=.92; CR^a^** **=0.94; AVE^b^** **=0.85)**			
	I feel there is meaning to my present and past life	0.93	
	I have a sense of continued self-development and growth	0.92	
	I have a positive attitude toward myself and life in general	0.91	
**Relaxation (α=.93; CR=0.95; AVE=0.87)**			
	Playing an open-world game makes me feel content	0.94	
	When I play an open-world game I feel relaxed	0.93	
	Playing an open-world games makes me feel stressed (reverse)	0.93	
**Cognitive escapism (α=.91; CR=0.94; AVE=0.84)**			
	Playing an open-world game helps me escape from my everyday problems	0.92	
	Open-world games help me forget the trouble and anxiety in my life	0.92	
	Playing open-world games makes me forget my worries	0.91	

^a^CR: composite reliability.

^b^AVE: average variance extracted.

### Study 3

#### Qualitative Study Design

Following our first qualitative and survey studies, we conducted a second qualitative study to further explore the specific elements of open-world games that may facilitate cognitive escapism. Specifically, we followed the same procedure as in qualitative study 1 (conducting semistructured, cross-sectional interviews using open-ended, predetermined questions and convenience sampling); we report the findings of qualitative study 3 in accordance with the Standards for Reporting Qualitative Research guidelines [29]. As part of this study, we conducted 15 semistructured in-depth interviews. As in study 1, we paid special attention to the importance of in-depth data collection to formulate and derive new theories. More specifically, and as in study 1, we ensured that questions were open ended. Furthermore, in an effort to minimize any potential bias, 2 trained RAs conducted the interviews, and any discrepancies were discussed. Moreover, as in study 1, the 2 RAs were trained not to guide the participants and their responses in any shape or form. The team of RAs and the entire research team was diverse in gender, age, and ethnicity; this ensured increased comfortability with participants during interviews and subsequent discussions.

#### Ethical Considerations

Ethics approval for the study was granted by the Department of Analytics, Marketing, and Operations at Imperial College London. All data were anonymized and kept confidential within the research team. Participants provided informed consent and had the right to withdraw from the study at any time.

#### Participants and Procedure

As in our qualitative study 1, we used a convenience sampling method and recruited study 3 participants using posters and handing out flyers to students on a university campus. The 2 eligibility criteria for taking part in this study were that participants were full-time students and that they had some personal experience playing an open-world game before. Over the course of 4 weeks (June 2024), 15 (n=6, 40% female; n=9, 60% male; mean age 23.53, SD 2.26 y) exploratory in-depth interviews were conducted with respondents, who had their student ID checked and had played an open-world game before (a trained RA asked some basic screening questions, including which specific open-world games they had play before and the basic content [story] of the games).

During the interviews, participants were invited to share their thoughts about the specific elements of open-world games that influenced their lives (“Which elements of open-world games influence how you feel in your daily life?” “How do they do so?” “Are there certain aspects of open-world games that you find most impactful in your daily life in terms of how they make you feel?” “Why do you enjoy playing open-world games, if at all?” “Are there any other elements of open-world games that you find noteworthy in terms of how they make you feel?” “Can you please share how these manage to do so?”).

As in study 1, interviews were again conducted in the alumni lounge and a campus café of a university and lasted between 75 and 135 minutes. Interviewees were informed about the confidentiality of their responses and were told that their participation would inform academic research. Each interviewee was thanked and received US $30 as compensation for their participation. Interviewees also had the chance to ask any questions they potentially had at the end of the study.

#### Qualitative Data Analysis

Following the same steps as in study 1, first each RA familiarized themselves with the data collected by transcribing the interviews and reading through the transcripts carefully. As a second step, the authors analyzed all the qualitative data to develop a holistic understanding of it. Specifically, we used open coding and descriptive phrases to name these codes to develop insights about the potential effects of open-world games on users. Interview data coding was conducted in duplicate by the authors, and any discrepancies were discussed and resolved. We again used the constant comparison technique and compared the newly collected interview data against the existing insights as we conducted more interviews to group conceptually linked data to reduce them to a set of meaningful concepts. Third, we finalized the key elements identified by grouping codes together under common ideas to derive overarching themes. Fourth, the themes were then revisited and reviewed by the researchers and cross-checked and compared with all the data collected to ensure that all key concepts had been captured by the key themes. Finally and fifth, we presented findings through a report that highlighted the key identified effects of open-world games with a brief description and exemplary quotes.

## Results

### Study 1: Results

The interview findings showed that open-world games’ affordance of cognitive escapism played a key role in their effect on players’ lives. Table 2 lists the representative quotes, which illustrate the extent to which open-world games were seen as providing cognitive escapism and players’ appreciation of cognitive escapism when playing. One interviewee noted the following:

Finally I’m feeling inner-peace. Exploring...wandering around...it allows me to forget my daily worries. It is liberating in a way. I don’t have to think about things that would otherwise upset me.

Respondent #8

Furthermore, the interview findings demonstrated the key role played by relaxation offered by open-world gaming. Respondents noted that reduced stress and enhanced relaxation were some of the key benefits of playing open-world games (Table 2). For instance, a respondent said the following:

There is this constant background noise. It stresses me out. It is exhausting and I get no respite. When I am playing I am less stressed. I breathe more easily. It calms me down. This is a true form of relaxation or being at peace with myself and the world moment.

Respondent #3

In addition to this, respondents shared their points about the extent to which open-world games impacted their mental well-being. One of the respondents, for instance, shared the following:

There are a million of things in my life that need taking care of. It never ends. [Game name] is something I look forward to. No matter how bad the day is, I know I can grow, explore, and keep going in [Game name]. Without it I would feel like something important is missing in my life. To keep me healthy and sane, oh, yes, spending time playing [Game name] is probably the way at the moment in my life.

Respondent #5

**Table 2:** Representative quotes of respondents regarding the key effects of open-world games.

Open-world games’ impact	Representative quotes
Cognitive escapism	“I need something that allows me to switch off at times. Constantly thinking about things drives me mad. Playing [Game name] allows me to switch off and not worry.” [Respondent #2] “It’s just always there. It never stops. Worrying about grades. Worrying about what others think. What’s going on in the world. It is exhausting to keep thinking about this all the time. I need some rest from all of this and this is what [Game name] gives me. It gives me space to not have to think about my daily worries all the time.” [Respondent #5] -“[Game name] is my form of mediation. There is no other time during the day when I feel at ease, no concerns, no distractions of thinking about various things. Yes, it really is the closest to mediation I can think of. It’s like a blessing and a refuge from stress.” [Respondent #6] “Exploring at my own pace, discovering new things, trying out new things...when I spend time playing [Game name] I feel absolutely at ease. [Game name] makes me feel less worried and less stressed out by all the things.” [Respondent #9] “What Nintendo does really well and I say Nintendo in particular is giving players a sense of peace and inner calm. Their Zelda: Breath of the Wild or Zelda: Tears of the Kingdom games are so positive. They make me feel I can escape the negativity in today’s world and be positive again. I can think of something else other than the madness that characterises social media and life in general these days.” [Respondent #10] “[Game name] is my home away from home. It is where I want to be. [Game name] gives me respite from all the stress. I need a break. I just need a break sometimes. [Game name] is the break I need.” [Respondent #13] “One could say it is a form of relief of stress and a way not to have to constantly fight the daily battle with issues. That’s what it is for me. [Game name] allows me to breathe easy, to just give it a rest and not overthink things all the time. That inner voice inside my head is finally silent. [Game name] gives me a chance to be calm inside my head.” [Respondent #14] “As a student I face constant pressure from peers, the programme, social media conversations, etc. [Game name] is the only place I have in my life that gives me peace from all these stressors. When I play [Game name] I worry less about these kind of things.” [Respondent #15] “[Game name] gives me a chance to lose myself in it. [Game name] is a break from all the things going on in my life right now.” [Respondent #16]
Relaxation	“It’s almost instant or after a short while of playing [Game name]. I start to feel at ease. I am less tensed. I am definitely less stressed out and worried about life.” [Respondent #1] “[Game name] is incredible. I feel more relaxed and rejuvenated after playing than after getting a massage. My body and mind are fully at ease. All the tension is gone.” [Respondent #3] “Primarily it is a soothing experience for me. [Game name] makes me feel at ease and less stressed out. It is relaxing for me.” [Respondent #4] “I can feel the difference it makes. Playing [Game name] makes me feel a lot calmer. My mind doesn’t feel so tense. And I can sense the effects on my body too. Even my muscles overall feel less tensed as a result.” [Respondent #5] “My muscles feel relief and so does my mind. [Game name] gives me relaxation. It relaxes me. I feel less stressed out and tense.” [Respondent #7] “I find it super relaxing. Everybody needs a Nintendo Switch. I recommend it wholeheartedly. Probably that’s the best investment you can make with your money today. Play Zelda on the Nintendo Switch and you can thank me later.” [Respondent #8] “It’s both. Both my body and mind feel relaxed. It’s definitely a relaxing experience.” [Respondent 10] “[Game name] calms me down. Playing [Game name] calms me emotionally. It soothes me. I feel at ease and some form of inner-peace. While it may sound a bit crazy, it’s real; [Game name] gives me inner-peace.” [Respondent #11] “[Game name] soothes my tired and exhausted mind.” [Respondent #12] “It’s relaxing and nothing is quite like it. I don’t have found anything that comes even close in terms of giving me a chance to breathe and be less tense, to feel loose and relaxed.” [Respondent #14] -“[Game name] alleviates my tension. It’s just soothing and relaxing to play. Very relaxing.” [Respondent #17]
Mental well-being	“[Game name] has made me a far more positive, satisfied person.” [Respondent #1] “Playing [Game name] has given me a new perspective in life. It’s not just about worries and anxieties. I can explore. I have a sense of achievement, of growth, of doing more with my life. [Game name] has had a very positive impact on my life. I feel a lot more content with what I have and who I am.” [Respondent #2] “[Game name] gives me a sense of fulfilment in my life. A sense of accomplishment and as a result I feel more satisfied and happy.” [Respondent #5] “Before [Game name] I often felt a loss of purpose and I felt there was just emptiness. [Game name] gave me a new perspective on life in general. There are still so many things I need to deal with as part of what’s going on but at least [Game name] gives me a mission, a quest, something different. To some others it may be trivial and meaningless but to me it holds deep meaning. I feel [Game name] gives me the strength I need to keep going in spite of everything.” [Respondent #8] “When I play [Game name] I feel a sense of direction a certain calling. [Game name] gives me a chance to explore and keep growing as a person. It is deeply satisfying. Overall it has a tremendous impact on my health and well-being on a day-to-day basis.” [Respondent #9] “Too many gaming companies go down the route of trying to lure people in and then rip them off. It’s like a casino. Stay away from such games. They are like social media, where your brain is reduced to clickbait and constant dopamine rushes that you dearly pay for. I love Nintendo for being so different and unique in the industry. Their open-world games are truly offering an immersive, positive sense of exploration and adventure. It reminds me of the times when I was a kid, exploring the countryside by myself. That was the best time of my life hands down. When I play Zelda: Breath of the Wild I feel like that kid again. There is nothing quite like it. I deeply enjoy and treasure that feeling.” [Respondent #10] “In an interesting way [Game name] makes me actually more at ease and accepting of my situation and who I am. I am happy with what I have got. I am happy with who I am and that’s also thanks to [Game name].” [Respondent #11] “It’s a rejuvenating experience. It clears my mind and I feel happier, more positive, less doom and gloom. There is so much negativity everywhere it is depressing. [Game name] allows me to think of other, more positive things in life. I feel less depressed and lonely. [Game name] gives me happiness.” [Respondent #13] “My mental health and well-being has improved significantly thanks to [Game name]. I feel a lot happier now than before.” [Respondent #14] “Life is depressing if it is only about solving problems. There will always be challenges and this and that to cope with. But life can’t only be about that. The freedom to explore, to grow, to learn, to see new places and do new things perhaps someone has not tried before, it’s just refreshing and that is exactly what [Game name] gives me.” [Respondent #16] “At one point I started to question the meaning of it all. Why even bother? What’s the meaning of all this? Of life? Is my life different from that of an ant? Does it make a difference to the universe? [Game name] just gives me a different perspective. I don’t have to know the answers to these questions. It’s OK. Life is OK. [Game name] gives me a sense of meaning, growth, development, exploration, wonder, appreciation, gratitude, relief, peace, beauty of the natural things around me. [Game name] has opened my mind to new things in my life or perhaps there were there but I could not see and appreciate them deeply. [Game name] has changed my life for the better.” [Respondent #17]

### Study 2: Mediation Analyses Results

The results for the mediation analyses showed that the direct effect of cognitive escapism on mental well-being was not statistically significant because 95% CIs included 0 (*P*=.43; Table 3). Notably, we found evidence for an indirect path of cognitive escapism through relaxation (standardized β=0.02; bootstrapped SE 0.009; 95% CI 0.0042-0.0390; Table 4). Specifically, the detailed results indicated that cognitive escapism afforded by open-world gaming had a significant positive impact on relaxation by the players (standardized β=.15; SE 0.04; *P*<.001; 95% CI 0.0695-0.2331; *F*_1,607_=13.21; *P*<.001, *R*^2^=0.02), which in turn had a significant and positive impact on players’ mental well-being (standardized β=.12; SE 0.04; *P*<.002; 95% CI 0.0445-0.2032, *F*_2, 606_=5.48; *P*=.004, R^2^=0.02). These results suggest that relaxation significantly mediates the effects of cognitive escapism on mental well-being (Table 4), supported by ANOVA results (Table 5).

**Table 3:** Mediation results.

	Relaxation				Mental well-being		
	Standardized coefficient (95% CI)	Coefficient (SE)	*P* value	Standardized coefficient (95% CI)		Coefficient (SE)	*P* value
Constant	—^a^ (3.11 to 3.84)	3.47 (0.19)	<.001	— (2.85 to 3.76)		3.30 (0.23)	<.001
Cognitive escapism	0.15 (0.07 to 0.23)	0.15 (0.04)	<.001	0.03 (–0.05 to 0.12)		0.03 (0.04)	.43
Relaxation	—	—	—	0.12 (0.05 to 0.20)		0.12 (0.04)	.002

^a^Not applicable.

**Table 4:** Total and direct effects of cognitive escapism on mental well-being.

	Effect (SE; 95% CI)	*P* value
Total effect	0.05 (0.04; –0.03 to 0.13)	.22
Direct effect	0.03 (0.04; –0.05 to 0.12)	.43

**Table 5:** Indirect and standardized indirect effects of cognitive escapism on mental well-being.

	Effect (bootstrap 95% CI)	Bootstrap SE
Indirect effects	0.02 (0.01-0.04)	0.01
Standardized indirect effects	0.02 (0.01-0.04)	0.01

### Study 3: Results

The findings of our second qualitative study showed which specific elements of open-world games participants noted as being effective in facilitating cognitive escapism, namely, (1) offering a sense of exploration, (2) giving players a chance to develop and experience mastery and skill, (3) offering a sense of positivity, and (4) providing a sense of purpose and meaning in life. One respondent noted the following:

It’s the freedom to roam around, to keep searching, to take the road less travelled...that makes me want to come back to [Game name]. It clears my mind and allows me to think freely, to entertain thoughts I’d otherwise not enjoy having. It is that liberty to go out there and explore the map, which then makes me feel free and lighter.

Respondent #1

Another respondent shared the following:

To be able to put in the hours but also see a reward, to see the character grow, do more, be more...it’s so satisfying. It’s empowering really and helps me free up my headspace and shut up that inner voice that tells me “no, you can’t do this or can’t do that.”

Respondent #6

Furthermore, as part of our interviews, 1 respondent indicated the following:

After a hard and difficult day I feel exhausted and empty. And The Legend of Zelda games are so positive. No matter how much trial and tribulation Link and Zelda have to go through, there is a positive side to it. An encouraging message. A path that opens up or is worth searching for and support to keep going. The positivity of the games such as Zelda go a long way. It truly helps me cope with the stress and challenges in my life. It gives me a chance to breathe easy if only for a while but also later in the day I look forward to [playing Zelda] and this positivity carries over and gets me through the day.

Respondent #2

In addition to this, a respondent shared the following:

Life sometimes feels meaningless. I mean why bother if our lives really don’t matter much to the universe than the life of an ant? Sure, some people think themselves more important than ants but to the cosmos really they may be the same. Sometimes I struggle because of this and playing [Game name] gives me a reason and something to look forward to, goals to reach, things to explore and conquer, to keep learning, and I enjoy the process, the journey. It may sound crazy but it is meaningful to me this journey. Let’s say I start of as Link in The Legend of Zelda: Breath of the Wild in the shrine of resurrection...I wake up...walk out of the shrine and see this vast world, free to explore, free to try, free to fail, free to keep going, growing...it actually is quite moving. As in life, you start of butt-naked. Well, they give you basic clothes but no armour, no shield, no bow and arrow...you gain all these by exploring the world on offer along the way. During the day and night I may be engaged in tasks that...who knows if they are meaningful or not but when I spend time in Hyrule I feel a sense of meaning. The game gives me purpose in life. It is a beautiful reason for me to go through some of the other things in life. It grounds me. It anchors me. Yes, it gives me meaning in life.

Respondent #3

Additional representative quotes for these specific elements of open-world games noted by the respondents are listed in Tables 6-9.

**Table 6:** Representative quotes for the theme of “offering a sense of exploration.”

Open-world game elements and description			Representative quotes
**Offering a sense of exploration**			
	Nonlinear gameplay allowing exploration and interaction at own pace	“It’s a very soothing experience to be able to enjoy and explore [Game name] and different paths within the game at my own pace.” [Respondent #1] “Sometimes it is super tedious to be asked or forced to follow a certain, very linear and predictable play in a game. It becomes a chore not a game anymore. I feel like I have to do certain things and simply follow instructions. That’s not what I want. Once I feel I feel boxed in with all these rules, it just loses its appeal. Being able to make my own decisions and draw my own path and make decisions on the fly as to what to explore next is priceless. This is where the exploration element comes in for me that gives me flow and losing myself in the exploration and journey itself.” [Respondent #5] “The moment I begin to feel it is all about pressing the right buttons at the right time I am out. Count me out. This is stress for me and quite the opposite that I need and seek in a game. I feel like a rat in a laboratory then asked to press various buttons at the right time. I don’t want to be that rat. Let me decide, give me the freedom to make a decision what step to take next in the game. I may regret it but at least I chose.” [Respondent #8] “You never know what’s around the corner. That’s why it is so magical. There may always be a surprise knocking on your door. Maybe you did not expect it but it surprised you and it put a smile on your face. I love those moments. That’s why life is magic. [Game name] does this really well. One moment you are fighting a mystical creature, and the next moment you are shield-surfing in the snowy mountains. Perfection. I feel like the time stops in those moments and my mind and body are rushed with feelings of complete calm.” [Respondent #10]	
	Immersive, detailed environments that offer a sense of freedom	“There is this immense open world that is out there to be explored. It is inviting me to keep looking, to take different turns and enjoy the twists in doing so. Each region has its own characteristic and I feel like I am on this grand adventure, conquering different parts of the map that I enjoy and find so liberating in many ways.” [Respondent #3] “From the majestic and vast, vast Hebra Mountains to the Wetlands of Lanayru and the scenic, leafy fields of the Akkala Highlands, all the way to the punishing heat of Death Mountain, and the sandy beaches of the Necluda Sea, and the extreme temperatures of the Gerudo Desert, and not to mention of course the Hyrule Field, and the Great Plateau, where all the story starts...I could go on, there is so much beauty, so much to freely explore. WOW! It truly is a world out there. It is special and I feel I want to be a part of it all. It is a part of me. That is what bliss feels like for me the freedom to explore these beautiful regions.” [Respondent #6] “One can easily spend weeks and weeks exploring the Hebra Mountains alone. C’mon that place is wild. It’s crazy. It’s so beautiful and rich in depth and full of adventure and danger lurking everywhere. Get your shield and get boarding on snow! You even get the world-champion tutoring you, when you find her. It’s just nuts. I completely forget about time when I play. When I want some quality me time this is it.” [Respondent #11] “There was this ad with someone on the bus driving home after work. This hit hard. I was like that’s me on the bus. I got [Game name] and I am grateful I did. Actually do you know that our planet Earth is beautiful? You wouldn’t believe it reading all the negative stuff on social media and so called news out there. It is difficult not to get depressed by it all. So here I was and I started to play, not exactly knowing what to expect, and it was such a thing of magic. There was adventure and a sense of exploration around each corner...it blew my mind and even now after months and months it makes me feel warm and fuzzy inside thinking about it. The world is so detailed and varied and just incredible what may explore in it. It is brilliantly done. Breath taking and so calming too with a chance to do something without the constant worry about stress and being anxious about this and that in my life. Wonderful.” [Respondent #15]	
	Vast and varied game worlds provide a chance for personal journey and growth	“Often I catch myself thinking I am the on the journey here. It’s me doing the exploring and growing.” [Respondent #4] “Through all this trial and tribulation, the frustrations of tackling some of the shrines that are really a pain in the neck to figure out and to find I have grown as a person. Patience. Self-Discipline. Kindness. Friendship. Dedication to the craft...yes, I have grown as a warrior not just Link.” [Respondent #8] “[Game name] is fabulous. Full of adventure and challenges and so many places and crafts to explore. Each place has hidden secrets. To unlock them one needs patience and a lot of grit. Getting lost in Hebra Mountains, c’mon, they are just so vast. It is wild. I learned a lot about myself when playing [Game name]. You think it is all about moving around Link but it’s not. It is about you as a player, as an explorer, of the captain of your own life and the adventures to unlock along the way.” [Respondent #10] “Did you know that Bushido means ‘the way of the warrior’? Courage and bravery but also politeness and kindness as well as a sense of justice and faithfulness are things I have considered when playing [Game name]. It has become a part of me and Link’s journey in many ways is my journey.” [Respondent #12]	

**Table 7:** Representative quotes for the theme of “offering a sense of mystery and skill.”

Open-world game elements and description			Representative quotes
**Offering a sense of mastery and skill**			
	Enhances feelings of satisfaction and competence by enabling players to overcome various challenges and struggles	“Finally I managed to get the master sword. What a moment. I waited for this moment so long! That’s the feeling you live for. Total bliss and total feeling of relief and peace.” [Respondent #3] “I often struggle with the fact that I seem to hit a wall. How to proceed in life? What to do next? It all seems so random and chaotic. Everyone is negative and deeply pessimistic, selfish, tribal, and self-obsessed in this world right now. It’s a welcomed escape for me to...well, learn new things in [Game name], see Link gain new skills, I learn new skills. I thought I would never manage to beat the four divine beasts but finally I did it. The first time I found a Korok seed, solved one of those puzzles...I will never forget those moments. They are staying with me.” [Respondent #6] “Mezza Lo Shrine was impossible to complete. It took me days and days and I couldn’t get it done. That buck always escaped. And you know what? I hit the wrong button! Hahaha, instead of the L button I kept spamming the R button. It was hilarious. What a hoot. It really brought home the message of never giving up. And in the end I did it. I completed that damn shrine. Hahaha. Heart in the mouth stuff. I felt I could manage it and do other things too. If I can manage the Mezza Lo Shrine I can overcome other challenges.” [Respondent #11] “Never look back too much unless it’s for gratefulness and learning that’s what [Game name] taught me. Nunquam retro as the old Latin saying goes. [Game name] threw challenges at me I never thought I’d manage ever. They were impossible. At least that’s what they looked like to me at the time. Funny how I managed in the end. I learned so much. The frustrations were huge at times. You know how it feels you try and try and try and it simply won’t work? Yes, that’s exactly how I felt. But then I did it. I was able to solve the riddles, unlock the paths, it was amazing.” [Respondent #13]	
	Enables players in their daily lives by offering a feeling of being capable and able to shape one’s environment or destiny	“What Link has shown me is that I can be more than a one trick pony. I still have got the moves and can put them to use in dangerous situations, facing obstacles and enemies. There is no reason to believe we cannot change anything. This enabled me in my life too. To know and to think one isn’t done yet. Life is not over until it’s over.” [Respondent #9] “I used to spend four to six hours on social media every day. It got to me in the end. It made me feel lonely, sad, anxious, depressed and deeply pessimistic about everyone and everything. It was a rabbit hole and I couldn’t get out. Utterly powerless and a sense of being at a loss that’s what it felt like day in and day out. [Game name] changed my life. It’s crazy. I felt peace and inner calm. I could play, nobody else, I played and that’s it. After months into the game I thought to myself ‘well, life isn’t too bad’. I can do this.” [Respondent #11] “I very much felt empowered by this. If I can conquer the different terrains of [Game name] I should be able to handle the other things here in life.” [Respondent #14] “It’s not this static thing. Absolutely it’s not the case that there is no response. There is. As the protagonist you can change the route of the story by the actions you take. This made me think about my own life and what I do and what I don’t do and how one step opens up other things and so on.” [Respondent #15]	

**Table 8:** Representative quotes for the theme of “offering a sense of positivity.”

Open-world game elements and description			Representative quotes
**Offering a sense of positivity**			
	Engaging and positive narratives provide encouraging emotional experiences	“Switching on the TV or even worse watching anything on the internet is saying hello go gloom and doom. The end is neigh. Pack your packs the world as we know it already ended and that’s what the internet is all about 24/7. Negativity rules in anti-social media. The sad part is how addictive it all is scrolling down reading the shocking, sad, outrageous things. It messes with your head and leaves you ultimately sucked dry and empty. I tell my friends ‘everyone needs a Nintendo Switch’. The positivity of [Game name] is what makes it for me. The story is so positive and keeps you positive.” [Respondent #2] “Well, it’s not really novel exactly the story...when you think about it, it’s that ancient good versus evil. But that’s OK. That story never gets old. It’s part of human existence. It’s what we are all about individually and as a species. We all struggle with it and it’s a part of us and who we are. So that’s all OK. Don’t be worried about it too much. Any story can be told in multiple ways. It can be sad and leave you anxious. It can be encouraging and make you feel stronger and more positive. Beating Calamity Ganon may be the goal but it’s the story and journey along the way that makes you more positive. Also it’s true genius that they allow you to fight Calamity Ganon over and over again without having to start a new game. That’s absolutely brilliant. I love the positive ending. It’s such a positive and encouraging story.” [Respondent #5] “I want more positivity in my life. [Game name] gives me that, which makes me feel better and at ease, less stressed out and worried about all sorts of things going on.” [Respondent #14]	
	Gives players a sense of belonging and makes them feel as though they have a home away from home	“I was travelling and I didn’t bring my console. So I couldn’t play [Game name] for a few weeks. And it’s like, I missed it. The moment I saw Hyrule Fields, I am like ‘I am back. I am home’. It was such a warm feeling.” [Respondent #1] “[Game name] is this place I belong. I feel I belong there and it’s part of me.” [Respondent #10] “[Game name] is so familiar to me, yet offering always something new. It’s so comforting in one way and giving adventure and exploration also.” [Respondent #12] “You can always go back to it. I know it is there for me when I need it. In many ways it is like a home, where you can enjoy, be yourself, not worry about the madness of the world. It’s home.” [Respondent #14]	

**Table 9:** Representative quotes for the theme of “offering a sense of purpose and meaning.”

Open-world game elements and description			Representative quotes
**Offering a sense of purpose and meaning**			
	Provides opportunities for introspection and self-reflection as part of gameplay	“Life can be so dull and always the same thing, the same old, the same loneliness, the same negative things, and so [Game name] is this break I need from all that. It gives me a chance to think about other things and...then I think about things that probably otherwise I wouldn’t have thought about at all.” [Respondent #3] “What does it say about me as a player and what does it mean for me if I don’t manage this I would ask myself quite often when playing [Game name]. But most often I wouldn’t think about anything in particular and that was the beautiful part. It’s quite rare not thinking about a zillion things at a time. Then I thought about my life and what I do and then I thought about what I want to explore. It allowed me to explore my own situation more deeply, which is rare.” [Respondent #7] “It can all appear as to have to meaning. My friends always say that it doesn’t matter. When the lights are switched off then that’s that. End of story. There could be more. What if there is more? In [Game name] the story does not end, why should my life end?” [Respondent #11] “Perhaps that’s what it’s all about? Maybe we are already in a game, in a simulation? Elon Musk thinks so. Why not? It could be real. It’s real. [Game name] is a story and I play it and I think how my story is going and how it will end maybe.” [Respondent #13]	
	Allows players to transcend the worries and stressors of daily life	“WOW! It’s the small things that kill me. You know, that email or that comment or this picture or that someone’s words...In the bigger scheme of things they are all, well, do they matter? In [Game name] I feel there are more important things than these petty small stuff to sweat. There are more to life and to see and enjoy.” [Respondent #4] “There is so much pressure from university. My parents are stressed out and I am stressed out. I don’t know how to cope. [Game name] finally allows me to forget about these worries and move on not being constantly bombarded with stressful things.” [Respondent #9] “I get worried so much and it’s hard to stop worrying. So [Game name] helps me breathe easy and not have to be worried all the time non-stop. I can think of other things.” [Respondent #13] “Hit the reset button I tell myself and it is not easy to do. It is easy to say but it is not easy to do. Everyone needs a little help I think. Everyone needs a bit of something else besides stress and worries and problems to be solved all the time. [Game name] is my reset button.” [Respondent #15]	

## Discussion

### Principal Findings

By examining how open-world games afford cognitive escapism, relaxation, and mental well-being, this study significantly contributes to the understanding of video games’ complex psychological impacts. Specifically, this research may inform game design, promoting features that maximize these benefits, and guide mental health professionals in using open-world games as therapeutic tools.

In brief, the findings of this study show that the cognitive escapism afforded by open-world games contributes significantly to relaxation and overall well-being among postgraduate students. By providing immersive environments that allow mental diversion, emotional relief, and meaning, these games can serve as valuable tools for enhancing psychological and emotional health. We invite future research to continue to explore these relationships to better understand how to maximize the therapeutic potential of open-world games while mitigating any potential negative impacts.

Taken together, this study on open-world games and their potential effects on cognitive escapism, relaxation, and well-being fills a significant gap in the current literature. By offering a detailed exploration of open-world games’ unique game characteristics, the findings of this study provide valuable insights into how digital environments may positively impact mental health and well-being, paving the way for more targeted and effective uses of video games in therapeutic settings.

### Comparison to Prior Work

Previous studies [9,15] demonstrated that casual gaming could reduce stress and improve mood, suggesting that video games can serve as effective tools for relaxation. This study builds on these findings by focusing specifically on open-world games and confirming that the freedom and autonomy provided by these games contribute to relaxation and mental rejuvenation.

While previous research has generally addressed the benefits of video games [19], this study focuses on the unique aspects of open-world games, confirming their significant positive impact on mental health in the context of postgraduate students. Future research should continue to explore these relationships, particularly the long-term effects and therapeutic potential of open-world games for managing stress and anxiety.

### Limitations and Future Research

This study has several limitations, which also offer promising avenues for future work. For instance, while in-depth interviews were conducted and open-world game players were surveyed as part of this research, the reliance on self-reported data is a limitation that is also an opportunity for future work. While self-reported data are valuable, they may be complemented by physiological or observational measures to provide a more comprehensive assessment. Future research that uses physiological measures to examine the effects of open-world gaming on mental well-being is richly deserving. Furthermore, arguably this study explored more of the immediate or medium-term effects of gameplay but was not able to measure possible long-term effects. Medium-term stress reduction and relaxation may not necessarily translate into sustained mental health benefits in the long term. Longitudinal studies are needed to assess how regular engagement with open-world games influences mental health over time. In addition, the relatively small R^2^ value in our quantitative analyses (study 2) may be due to potential confounding variables that could be considered to further strengthen the power of the analysis. For example, prior research has highlighted the anxiety and stress that social media and university studies may cause [40-43] as well as the positive effects of gamification [44-48], hope and well-being benefits [49,50], and the role of gaming in enhancing critical skills and knowledge, which contribute to individuals’ connections and well-being in life [47,51-57]. Future research examining open-world games’ influence on people’s lives and potential negative effects as well as their ability to contribute to overall happiness and life satisfaction in the long term is richly deserving.

### Summary

This study investigates the impact of open-world games on cognitive escapism, relaxation, and well-being among postgraduate students. Open-world games, known for their expansive, immersive environments and player autonomy, offer unique opportunities for players to escape from real-world stressors. The study used a mixed methods approach, conducting 32 in-depth exploratory interviews and surveying 609 postgraduate student players to assess the effects of these games. The findings of this study show that players reported significant cognitive escapism, with the ability to divert their thoughts from real-life concerns to engaging in-game activities. Enhanced well-being was observed, with players reporting increased relaxation, improved mood, and greater psychological satisfaction.

### Conclusions

This study indicates that open-world games significantly contribute to cognitive escapism, relaxation, and overall well-being. Specifically, these games’ immersive environments and player autonomy play a crucial role in reducing stress and enhancing mental health. Open-world games could be used as therapeutic tools for stress and anxiety management, offering a cost-effective and accessible method to improve mental health. Developers should consider incorporating features that promote relaxation and cognitive escapism to enhance the well-being of players. The finding that open-world games may enhance people’s well-being through enhanced escapism and relaxation is not trivial, given the growing evidence that other forms of entertainment, such as traditional social media, contribute to adolescent anxiety and depression [58-60]. We invite future research to build on the findings of this study and examine the role of open-world games in people’s lives further.

## Appendix

Multimedia Appendix 1 Strengthening the Reporting of Observational Studies in Epidemiology (STROBE) checklist for cross-sectional studies.

## Abbreviations

RA: research assistant
STROBE: Strengthening the Reporting of Observational Studies in Epidemiology

## Data Availability

The datasets generated during and analyzed during this study are available from the corresponding author on reasonable request.
